# Correspondence

**Published:** 1872-05

**Authors:** 


### CORRESPONDENCE.

EDITORS COMPANION.—We are passing through a fearful epidemic in this county, and from what I can learn, it is not confined to this county or State, but has been doing its work of destruction in other parts of our country. The subjects, in the main, are children, from one to ten years of age, and old persons, from fifty years and upwards. They are attacked with a nervous chill, followed by severe pain in the head and back of the neck. A majority of the well marked cases become insensible in a few hours, restless, talking incoherently, and tossing from side to side, and at times frantic as maniacs, but little fever, pulse feeble, but at times quite frequent, pupils of eyes much dilated, apparent loss of sight and hearing, head thrown back, with great pain on motion, and about half the cases thus *severely* attacked die within five days; less severe cases linger for ten or twelve days, and then die of exhaustion, or recover slowly. The disease has been called by some physicians "Meningitis," which I think, is a misnomer. Most, and perhaps, I may say, all the cases I have seen, have had, beside pain in head and neck, an inability to use one or both arms without pain, or one or both legs, or all the limbs together, and this seems to be the out-cropping of the disease, and evidently indicates the true character of the disease, namely: Rheumatism or Neuralgia of the brain and spinal cord, or their coverings. I have, for the want of a better name, called it rheumatic fever, from a shock of the nervous centres.

The cause of this epidemic, in my opinion, is quite apparent, viz: The loss of animal heat, or want of power to supply the demand of the requisite amount of it, to insure health. The loss of all animal heat in man, is death; the want of the power to supply the demand, produces disease that will lead to death if that power is not restored.

It is well known that there has been a greater aggregate amount of cold since November last, than any winter for the past thirty or forty years, and this too, has been general, and the people were not acclimated to the season; as well might we expect to have our usual health, if we should be transported, *en masse*, ten degrees north to spend a winter season. The ability to supply the requisite amount of animal heat, this cold season, has failed, and the feeble have fallen a prey to the demand. And here I come to my old theory. As in chills and fever, from loss of animal heat and vigor to supply the demand in sudden changes from heat to cold, and damp exhalations from the earth, in a country of rich, alluvial soil, readily emitting the cold, damp vapor, the mercury rising and falling 30° to 40° in the twenty-four hours. So, this long, cold winter and spring has so exhausted the nervous and physical system and its energies, that the feeble and exposed could no longer resist. A nervous shock is received, and rheumatism attacks the brain and spinal cord and their coverings, and thus, in my opinion, has the epidemic in question, been produced, and not by the poisoning of the blood by *miasmatic* influences, either in the present epidemic or in common chills and fever, as is the old theory.

The existing causes of the present epidemic, may be any exhausting exertion of body or mind, or any shock to the system, mental or physical. The more nervous and excitable, the more liable.

As to treatment, my plan has been to treat it as rheumatism. Warm baths, warm fomentations to the parts affected, soothing anodynes to allay nervous excitement and procure rest; stimulating and nourishing drinks, as milk, milk-punch, animal broths, and such other drinks as the patient may desire.

Very little medicine seems to be required, or is of but little use.

JOHN H. WEIR.

Edwardsville, Ill., April 1st, 1872.

---

CRYSTAL SPRINGS, MISS., Feb. 28th, 1872.

*Messrs. Powell & Goldsmith.*

GENTLEMEN.—I wish to report a case, which, to me at least, is something rare. It is this: Mrs. S—, a lady aged about twenty-five, having borne one child, and aborted once, again became pregnant. Sometime in the month of January last, she miscarried with a two months child. Every portion of its body perfectly developed for its age, except the head, there was none, and the neck seemed a smooth, round stump. This is what her mother told me, there being no physician present at the time. In a day or two after the accident, she was apparently well, and moved from her neighborhood to another about five miles distant. Five weeks after the accident, she was taken, on Sunday night, with profuse hemorrhage, which recurred at frequent intervals until Wednesday evening following. Wednesday night she was attacked with a severe spell of "flooding," and sent for a young physician in the neighborhood, who visited her and attempted, unsuccessfully, to arrest it. Early Thursday morning I was sent for in consultation. On my arrival I found the lady white as a sheet, extremely restless, vomiting occasionally, with a small, rapid, wiry, intermitting pulse. Indeed, every indication of a patient dying from loss of blood. Upon inquiry, I elicited the above history. I asked her attendant if he had examined the patient, on his replying in the negative, I at once commenced an examination per vaginum, and discovered the placenta in the mouth of the womb, which I immediately removed, all but a small portion, about the size of my thumb. Upon the removal of the placenta, which was about the size of my fist, perfectly preserved in a sound and healthy state, without the least offensive scent, the hemorrhage ceased. After several fruitless attempts to remove the remaining portion of the placenta, I put her on fluid extract ergot and carb. ammonia, every two hours, and left.

Upon my visit next morning, I found the portion of placenta had passed away, and the womb had thoroughly contracted, and receded to its natural position. She was placed upon tonics and nutritious diet, and up to this writing—two weeks—she is very near recovered. Respectfully,

THEODORE P. LOCKWOOD, M. D.

---

